# An Outcome Measure of Functionality and Quality of Life in Patients With Cervical Myelopathy

**DOI:** 10.5812/ircmj.8102

**Published:** 2014-06-05

**Authors:** Parisa Azimi, Omidvar Rezaei, Ali Montazeri

**Affiliations:** 1 Department of Neurosurgery, Shahid Beheshti University of Medical Sciences, Tehran, IR Iran; 2 Mental Health Research Group, Health Metrics Research Centre, Iranian Institute for Health Sciences Research, The Academic Center for Education, Culture and Research, Tehran, IR Iran

**Keywords:** Validation Studies as Topic, Spinal Cord Diseases, Iran

## Abstract

**Background:**

Cervical spondylotic myelopathy (CSM) is a common cause of significant clinical morbidity. The Japanese Orthopedic Association Cervical Myelopathy Evaluation Questionnaire (JOACMEQ) is a measure of health-related quality of life in these patients.

**Objectives:**

This study aimed to cross-culturally translate and validate the JOACMEQ in Iran.

**Patients and Methods:**

This study was a prospective clinical validation one. Forward-backward procedure was applied to translate the questionnaire from English into Persian. The translation and cross-cultural adaptation were performed in accordance with the published guidelines. A sample of patients with CSM was asked to respond to the questionnaire at two times: providing preoperative and postoperative assessments (6 months follow-up). To test the reliability, the internal consistency was assessed by Cronbach α coefficient and the validity was assessed by convergent validity. Responsiveness to change was also assessed comparing patients’ preoperative and postoperative scores.

**Results:**

All 87 patients completed the questionnaire. The Cronbach α coefficient for the JOACMEQ at preoperative and postoperative assessments ranged from 0.71 to 0.82 indicating a good internal consistency for the questionnaire. In addition, the correlation of each item with its hypothesized subscale of the JOACMEQ showed satisfactory results suggesting that the items had a substantial association with their own subscales. Further analysis also indicated that the questionnaire was responsive to change (P < 0.001).

**Conclusions:**

In general, the findings suggest that the Persian version of the JOACMEQ is a reliable and valid measure of functionality and quality of life evaluation among Iranian patients suffered from CSM.

## 1. Background

Cervical spondylotic myelopathy (CSM) is a progressive degenerative disease of the cervical spine. It is usually a chronic and progressive disease. As people grow older, the prevalence of CSM increases (1). Thus, the assessment of functionality and pain in those who suffer from this disease is an important part of clinical practice. As such, the Japanese Orthopedic Association scoring system (JOA score) is a well-known instrument to assess functionality in this population. The JOA score was first established by a committee of the JOA chaired for assessment of cervical myelopathy (2-4). The JOA revised the JOA score for cervical spondylotic myelopathy and developed a new disease-specific, patient-oriented outcome measure. The Japanese Orthopedic Association Cervical Myelopathy Evaluation Questionnaire (JOACMEQ) has been validated as an outcome measure in these patients (5-9) and was used by Japanese investigators (10-12).

## 2. Objectives

The aim of this study was to translate the JOACMEQ into Persian, and then to use and validate the questionnaire in studies of functionality and quality of life in CSM affected patients in Iran. 

## 3. Patients and Methods

### 3.1. Questionnaire

The Japanese Orthopedic Association Cervical Myelopathy Evaluation Questionnaire (JOACMEQ) is a revised version of the Japanese Orthopedic Association score (JOA). It was developed for the purpose of evaluating cervical myelopathy disorders (5-9). It is a self-administered, disease-specific tool and contains 24 items subdivided into five subscales: lower extremity function (5 items), quality of life (8 items), cervical spine function (4 items), bladder function (4 items), and upper extremity function (5 items). The score for each subscale ranges from 0 to 100 and higher scores indicate better conditions (7, 13). A user’s guide and scoring manual for the JOACMEQ are available (13).

### 3.2. Translation

The 'forward-backward' procedure was applied to translate the JOACMEQ from English into Persian. Two general practitioners translated the questionnaire into Persian, and then the translated text was translated backward into English by a health professional and a professional translator. After a careful review, cultural adaptation, and few changes, a provisional Persian version of the questionnaire was provided.

The provisional version was tested on a number of patients in order to establish that patients could understand this version and that the questions measure what they are intended to measure. Most patients correctly understood the questionnaire. Their general comments about the difficulty of completing the questionnaire or understanding the texts were asked, and after a consensus by authors, the final version was developed.

### 3.3. Patients and Data Collection

A cross-sectional study was conducted and the final draft of the Persian version of the JOACMEQ was administered to a convenient sample of newly diagnosed CSM patients attending the neurosurgery clinic of a large teaching hospital in Tehran, Iran during May 2008 to November 2011. There were no restrictions on patient selection with regard to types of CSM, age or other characteristics. The exclusion criteria were prior cervical spine surgery and spinal anomalies.

It was estimated that a sample of 72 patients (at least 3 patients per item) would be enough to carry out psychometric tests as recommended (14). A trained neurosurgery resident collected the data during one complete calendar year. Patients were assessed at two points in time: preoperative and postoperative (6 months follow-up).

### 3.4. Statistical Analysis

#### 3.4.1. Reliability

to test the reliability, the internal consistency of the questionnaire was measured using Cronbach α coefficient and α ≥ 0.70 was considered satisfactory (14).

#### 3.4.2. Validity

Validity was assessed performing item-scale correlations. Correlations were calculated using Pearson’s correlation coefficient (*r*). It was expected that item scores would correlate higher with its own hypothesized scale than other scales. Correlation values of 0.40 or above were considered satisfactory (*r* ≥ 0.81-1.0 as excellent, 0.61-0.80 very good, 0.41-0.60 good, 0.21-0.40 fair and 0.20 poor) (14).

#### 3.4.3. Responsiveness to Change

Responsiveness as a psychometric property of the questionnaire was also assessed. As such, patients’ preoperative and postoperative scores were compared using the paired t-test to examine whether JOACMEQ could capture the change after the intervention (surgery).

### 3.5. Ethics

The Ethics Committee of Shahid Beheshti University of Medical Sciences approved the study (dated 05.2008-565523). All patients gave their informed consent after receiving both written and oral information about the project.

## 4. Results

A total of 96 patients were enrolled in the study. Of them, 87 patients completed the questionnaire and 9 patients were excluded due to prior cervical spine surgery or spinal anomalies. Table 1 shows the characteristics of the CSM patients and their scores on the JOACMEQ. The mean age of patients was 50.3 (SD = ± 10.2) years; most were married (73.6%), and had completed primary or secondary education (69.0%).

The internal consistency of the JOACMEQ as measured by the Cronbach α coefficient ranged from 0.72 to 0.80 at preoperative assessment and from 0.71 to 0.82 at postoperative evaluation indicating a satisfactory result. Table 2 presents the results.

Validity of the JOACMEQ was examined using item-scale correlations. Table 3 presents the item-scale correlation matrix between each item and the five subscales of the JOACMEQ. All correlations between items and their hypothesized scales showed satisfactory results suggesting that the items had a substantial association with their own subscale. Pearson correlation coefficient exceeded the recommended level (*r* ≥ 0.40) ranging from 0.49 (Q2-4) to 0.80 (Q1-8). Responsiveness to change was assessed by paired t-test. In all instances, the JOACMEQ could detect changes after the intervention (surgery) indicating improvements in all subscales as expected. Table 4 shows the results.

**Table 1.** The Characteristics of the Study Sample (n = 87) ^a^

-	Values
Age Groups, y	
Mean ± SD	50.3 ± 10.2
Range	24-79
**Gender**	
Male	39 (44.8)
Female	48 (55.2)
**Educational status**	
Illiterate	14 (16.1)
Primary School	38 (43.7)
Secondary School	22 (25.3)
College/university	13 (14.9)
**Marital status**	
Single	15 (17.2)
Married	64 (73.6)
Divorced/Widowed	8 (9.2)
**Type of disease**	
Cervical herniated disk	50 (57.5)
Cervical spinal stenosis	37 (42.5)

^a^ Data are presented as No. (%).

**Table 2.** Descriptive Statistics

-	Number of Items	Cronbach α Coefficient ^a^ (Preoperative)	Cronbach α Coefficient ^a^ (Postoperative)	Floor Effect (%)	Ceiling Effect (%)
**Lower extremity function**	5	0.75	0.82	0	0
**Quality of life**	8	0.80	0.79	1.140	0
**Cervical spine function**	4	0.78	0.77	0	0
**Bladder function**	4	0.74	0.76	0	0
**Upper extremity function**	5	0.72	0.71	0	0

^a^ A value of 0.70 or above indicates adequate reliability.

**Table 3.** Item-Scale Correlation Matrix for the Five JOACMEQ Subscales ^a^

Items (Item Number)	Lower Extremity Function	Quality of Life	Cervical Spine Function	Bladder Function	Upper Extremity Function
**Can you walk on a flat surface? (Q1-4)**	0.64	0.18	0.18	0.16	0.66
**Can you stand on either leg without holding onto something? (Or the need to support yourself). (Q1-5)**	0.63	0.24	0.25	0.20	0.14
**Do you have difficulty in climbing up the stairs? (Q2-2)**	0.51	0.26	0.28	0.22	0.24
**Do you have difficulty with one of the following: bending forward, kneeling or stooping? If you have difficulty with one of them, how difficult is it? (Q2-3)**	0.52	0.32	0.19	0.33	0.16
**Do you have difficulty walking more than 15 minutes? (Q2-4)**	0.49	0.55	0.27	0.10	0.26
**How is your present health condition? (Q2-1)**	0.24	0.59	0.17	0.24	0.32
**Have you been unable to do your work or ordinary activities as well as you would like? (Q2-5)**	0.22	0.68	0.19	0.31	0.24
**Has your work routine been hindered because of the pain? (Q2-6)**	0.18	0.70	0.24	0.14	0.25
**Have you felt discouraged and depressed? (Q2-7)**	0.27	0.68	0.23	0.19	0.12
**Do you feel exhausted? (Q2-8)**	0.24	0.59	0.27	0.27	0.24
**Have you felt happy? (Q2-9)**	0.31	0.51	0.23	0.26	0.14
**Do you think you are in decent health? (Q2-10)**	0.24	0.62	0.18	0.31	0.22
**Do you feel your health will get worse? (Q2-11)**	0.14	0.69	0.26	0.33	0.27
**While in the sitting position, can you look up at the ceiling by tilting your head upward? (Q1-10)**	0.18	0.26	0.55	0.14	0.26
**Can you drink a glass of water without stopping despite the neck symptoms? (Q1-11)**	0.30	0.19	0.74	0.16	0.18
**Can you look at your feet when you go down the stairs? (Q1-12)**	0.27	0.35	0.78	0.25	0.67
**While in the sitting position, can you turn your head toward the person who is seated to the side but behind you and speak to that person while looking at his/her face? (Q1-13)**	0.19	0.31	0.73	0.28	0.21
**Do you have urinary incontinence? (Q1-6)**	0.32	0.14	0.16	0.67	0.20
**How often do you go to the bathroom at night? (Q1-7)**	0.14	0.26	0.24	0.76	0.21
**Do you have a feeling of residual urine in your bladder after voiding? (Q1-8)**	0.22	0.10	0.22	0.80	0.25
**Can you initiate (start) your urine stream immediately when you want to void? (Q1-9)**	0.24	0.27	0.18	0.69	0.20
**Can you fasten the front buttons of your blouse or shirt with both hands? (Q1-1)**	0.25	0.14	0.09	0.24	0.78
**Can you eat a meal with your dominant hand using a spoon or a fork? (Q1-2)**	0.11	0.26	0.15	0.28	0.69
**Can you raise your arm? (Answer for the weaker side.) (Q1-3)**	0.18	0.19	0.19	0.32	0.58

^a^ Pearson correlation (*r*) equal to or greater than 0.40 was considered satisfactory. (Correlation ≥ 0.81-1.0 as excellent, 0.61-0.80 very good, 0.41-0.60 good, 0.21-0.40 fair, and 0.0-0.20 poor) (14).

**Table 4.** Responsiveness to Change as Measured by the JOACMEQ ^a^

	Preoperative	Postoperative	P Value ^b^
**Lower extremity function**	41.3 ± 24.3	69.7 ± 11.2	< 0.001
**Quality of life**	32.4 ± 25.6	56.1 ± 7.6	< 0.001
**Cervical spine function**	51.1 ± 29.3	76.9 ± 9.3	< 0 001
**Bladder function**	61.3 ± 19.3	79.3 ± 7.3	< 0.001
**Upper extremity function**	48.4 ± 15.4	59.1 ± 12.4	< 0.001

^a^ Data are presented as mean ± SD.

^b^ Derived from paired t-test samples.

## 5. Discussion

The results of the present study showed that the Persian version of the JOACMEQ is a reliable measure to evaluate functionality and quality of life in Persian-speaking patients with cervical myelopathy. As noted by its authors the JOACMEQ measures five factors and the score for each factor should be interpreted independently. The total score for the JOACMEQ was considered meaningless (7, 13).

The Cronbach α for the Persian version of the JOACMEQ exceeded the recommended threshold suggesting that the Persian version of the questionnaire has satisfactory internal consistency. However, our approach for testing reliability of the JOACMEQ was different from the pioneering designers of the questionnaire. According to their report, each patient was interviewed twice at an interval of 4 weeks and then, the reliability of the questionnaire was evaluated by determining the extension of the weighted kappa coefficient. They reported that the weighted kappa was more than 0.50 for all but one item, which was 0.49 (7).

To the best of authors’ knowledge, the Persian version of the JOACMEQ is the only condition-specific outcome measure for patients with CSM that was undergone psychometric evaluation in Iran. In addition, as far as we know, this research is the first attempt in the literature for translating and validating the JOACMEQ.

According to our results, this instrument seems to be a reliable and valid outcome measure for functionality and quality of life in patients with cervical myelopathy in Iran, and perhaps it could be validated in other languages to compare the results of possible upcoming studies. The JOACMEQ offers an effective method of evaluation for quality of life (12) and as suggested it is likely that the JOACMEQ succeeds and becomes a global standard to evaluate outcomes in patients with cervical myelopathy (6).

We carried out a number of limited tests to perform this validation study. In future, it might be necessary to perform other tests to establish stronger psychometric indexes for the JOACMEQ. Perhaps one might argue why the authors did not perform explanatory factor analysis (EFA) and confirmatory factor analysis (CFA) to ensure that the questionnaire fits to the data.

In response, we should indicate that the questionnaire and its scoring procedure does not allow performing such analyses. For instance, there are items that should be included in different subscales. In addition, when calculating scores for subscales, all items should be multiplied by a given number. The scoring manual could be found in Appendix 1. Finally, we state that even the designers of the questionnaire did not perform any of the above analyses.

The findings from this preliminary validation study indicate that the Persian version of the JOACMEQ is a reliable and valid instrument for measuring functionality and quality of life in patients with cervical myelopathy.

## Appendix:

Scoring manual for the JOACMEQ:

Cervical spine function;

(Q1-10 × 20 + Q1-11 × 10 + Q1-13 × 15 + Q1-12 × 5 − 50)

Upper extremity function;

(Q1-12 × 5 + Q1-1 × 10 + Q1-2 × 15 + Q1-3 × 5 + Q1-4 × 5 − 40) × 100 ÷ 95

Lower extremity function;

(Q1-4 × 10 + Q1-5 × 10 + Q2-2 × 15 + Q2-3 × 5 + Q2-4 × 5 − 45) × 100 ÷ 110

Bladder function;

(Q1-6 × 10 + Q1-7 × 5 + Q1-8 × 10 + Q1-9 × 5 − 30) × 100 ÷ 80

Quality of life;

(Q2-1 × 3 + Q2-5 × 2 + Q2-6 × 2 + Q2-7 × 5 + Q2-8 × 4 + Q2-9 × 3 + Q2-10 × 2 + Q2-11 × 3 − 24) × 100 ÷ 96
